# Influence of the Compensating Cation Nature on the Water Adsorption Properties of Zeolites

**DOI:** 10.3390/molecules25040944

**Published:** 2020-02-20

**Authors:** Zakaria Tahraoui, Habiba Nouali, Claire Marichal, Patrice Forler, Julien Klein, T. Jean Daou

**Affiliations:** 1 Department Institut de Science des Matériaux de Mulhouse IS2M, Université de Haute Alsace (UHA), CNRS, UMR 7361, F-68100 Mulhouse, France; zakaria.tahraoui@uha.fr (Z.T.); habiba.nouali@uha.fr (H.N.); claire.marichal@uha.fr (C.M.); 2 Université de Strasbourg, 67081 Strasbourg, France; 3 APTAR CSP Technologies, 9 rue de Sandholz, 67110 Niederbronn-Les Bains, France; patrice.forler@aptar.com (P.F.); julien.klein@aptar.com (J.K.)

**Keywords:** zeolite, LTA-type zeolite, FAU-type zeolite, cationic exchange, magnesium, lithium, water adsorption

## Abstract

The influence of the compensating cation (Na^+^, Li^+^, Mg^2+^) nature on the water adsorption properties of LTA and FAU-type zeolites was investigated. Cation exchanges were performed at 80 °C for 2 h using 1 M aqueous solutions of lithium chloride (LiCl) or magnesium chloride (MgCl_2_). XRF and ICP-OES analyses indicate that the cation exchange yields reach values between 59 to 89% depending on the number of exchange cycles and the nature of the zeolite and cation, while both zeolites structures are preserved during the process, as shown by XRD and solid state NMR analyses. Nitrogen adsorption-desorption experiments indicate a higher available microporous volume when sodium cations are replaced by smaller monovalent lithium cations or by divalent magnesium cations because twice less cations are needed compared to monovalent cations. Up to 15% of gain in the available microporous volume is obtained for FAU-type zeolites exchanged with magnesium cation. This improvement facilitates the adsorption of water with an increase in the water uptake up to 30% for the LTA and FAU type zeolites exchanged with magnesium. These exchanged zeolites are promising for uses in water decontamination because a smaller amount is needed to trap the same amount of water compared to their sodium counterparts.

## 1. Introduction

The adsorption of water by porous solids is important for many applications which require capture and release of water such as electric dehumidifier, adsorption heat pumps (AHPs), alcohol/organic solvent dehydration, etc. One of the most promising AHPs technologies in this context is based on the evaporation and consecutive adsorption of water, under specific conditions. The first prototypes of adsorption heat pumps/cooling used adsorbent beds made of loose zeolite grains [1,2]. The water content in natural gas is also considered a critical concern because it can cause corrosion and hydrate formation, ultimately leading to pipeline blockage [3,4]. Several strategies were used to remove water vapor from gas streams involving supply a solid or liquid desiccant, membranes, refrigeration, supersonic methods and so on [5,6], but one of the most interesting strategies remains the use of a solid porous bed in which a porous desiccant with effective properties like high surface area, adsorption capacity, mechanical strength as well as being inexpensive, non-corrosive/toxic and chemically-inert serves for removing water vapor from a gaseous mixture [7,8]. Moreover, the need for moisture removal technology is also becoming important to improve the quality and safe storage of processed foods and moisture sensitive materials [9,10,11]. In daily human life relative humidity is also an important factor as it affects the health. Highly humid environments provide favorable environments for fungi, harmful bacteria and house dust mites to grow, and destroy the heat-humidity balance of the human body, etc. [12]. Therefore, the demand for controlling the humidity and development of high efficiency sorbent technology has led to a great interest in new porous materials, especially microporous materials [13].

A variety of porous materials (zeolites, metal organic frameworks, clays, carbon-based adsorbents, organic polymers) have been explored for all these applications, but still it remains a challenge to find low cost high performance materials combining high water uptake, precise operational pressure range control, recyclability, stability, etc. [14,15,16]. Zeolites are widely used for molecular decontamination due to their high adsorption properties and their thermal, chemical and mechanical stabilities. Zeolites are crystalline aluminosilicates with a 3-dimensional, open anion frameworks consisting of oxygen-sharing SiO_4_ and AlO_4_^-^ tetrahedra [17,18,19]. Each silicon ion has its +4 charge balanced by four tetrahedral oxygens, and the silica tetrahedra are therefore electrically neutral. Each alumina tetrahedron has a residual charge of −1 since the trivalent aluminum is bonded to four oxygen anions. Therefore, each alumina tetrahedron requires a +1 charge from an extra-framework cation in the structure to maintain electrical neutrality [18]. These cations are usually sodium ions that are present when the zeolite is synthesized [20]. Sodium ions can be easily exchanged by other mono- or divalent cations. Several studies have mentioned the major role that these cations (Na^+^, K^+^, Li^+^, Mg^2+^, Ca^2+^, Zn^2+^, Mn^2+^) can play in increasing the affinity between the adsorbates and the adsorbents or in modifying the separation properties of the zeolites [16,17,21,22,23].

In addition, it is well known that aluminosilicate zeolites containing compensating cations in their framework show a high hydrophilic character (especially the ones with low Si/Al ratio) which gives them a strong affinity towards water. The most commonly employed zeolites for water adsorption in industry are 3 Å (KA) and 4 Å (NaA) zeolites (LTA-type zeolite) and 13× (NaX) zeolite (FAU-type zeolite) [24,25,26,27,28,29,30]. FAU-type zeolites are one of the main components of cracking catalysts at industrial scale due to their structure. Their pore structures are composed of supercages, with a free diameter of 11.6 Å, interconnected through circular 12-member-ring (MR) apertures with a diameter of 7.4 Å [31]. The aluminosilicate framework of zeolite A (LTA-type) can be described in terms of two types of polyhedra, one being a simple cubic arrangement of eight polyhedra (double 4-rings); the other being a truncated octahedron of 24 tetrahedra also named a β-cage. In LTA, sodalite cages are joined via double 4-rings, creating an α-cage in the center of the unit cell. Alternatively, the framework can be described as a primitive cubic arrangement of α-cages joined through single 8-rings [32]. Zeolite A has a three-dimensional pore system and molecules can diffuse in all three directions in space by moving across the 8-ring windows of about 0.42 nm diameter that connect the cavities. The size of the pore openings depends on the size of the charge compensating cations. Normally, zeolite A is synthesized in the Na-form which has a pore opening of about 0.4 nm. The sodium cations can then be exchanged, thereby tuning the size of the pore openings.

All reported works agree that water adsorption in these zeolites is mainly directed by interactions between water, charge compensating cations and the zeolite framework. Depending on the charge (mono- or divalent cations) and the kinetic diameter of the compensating cation, the available microporous volume and the accessibility to the micropores are modified which lead to different adsorption behaviors and adsorption capacities [25]. Although, LTA and FAU-type zeolites show attractive adsorption uptake and high water affinity, their global performances regarding water adsorption are still not optimal, mostly due to the nature, size and affinity to water molecules of the compensating cations. Therefore there is still considerable need for improving the zeolite adsorbents for this targeted application. 

In this work, LTA-type and FAU-type zeolites provided by APTAR CSP Technologies were exchanged using MgCl_2_ and LiCl aqueous solution. The cation choice was made to have the smallest monovalent and divalent cations in order to maximize the available microporous volume. The prepared samples were then fully characterized and their adsorption performances were systematically evaluated by comparing their nitrogen and water adsorption isotherms.

## 2. Results

### 2.1. X-ray Fluorescence

The chemical composition of the raw and magnesium exchanged zeolite samples were determined by X-Ray Fluorescence analysis and the results are reported in Table 1. The framework of LTA zeolite consists of strictly alternating silicon and aluminum atoms leading to a Si/Al ratio of 1, the minimum allowed by the Lowenstein’s rule [33] which forbids two aluminum tetrahedrons to be linked. The FAU-type structure follows the same rules but since less aluminum atoms are present, more alternation between silicon and aluminum can be achieved with here an expected Si/Al ratio under 1.5 for X FAU-type and >1.5 for Y FAU-type zeolite. The chemical analysis in Table 1 shows that the Si/Al ratio obtained is around 1 for the raw LTA-type zeolite and around 1.20 for the raw FAU-type zeolite, in agreement with the expected value from the literature [34,35]. The concomitant sodium loss and magnesium increase as the cationic exchange steps progress is an indication of the successful exchange process (Table 1). Slight variation of the Si/Al ratio is observed as the cationic exchange steps progress pointing towards negligible aluminum extraction (See Section 2.4). It is worth to note that the cations/Al ratio in the raw LTA-type and FAU-type zeolites samples is slightly greater than 1, probably due to the slight excess of sodium on the surface compensating the negative charge due to the presence of defects (see Section 2.4).

The overall charge ratio (Na/Al + 2 Mg/Al) required in order to compensate the negative charges generated by the presence of aluminum atom is after exchanges greater than the one observed for the raw material. 

This cation charge excess observed for LTA-type and FAU-type zeolites could be attributed to the presence of sodium or magnesium cations at the surface of the zeolite, near negative charges generated by defects, or/and by chloride ions detected for LTA-type samples after cationic exchange. Another possible explanation is that a layer of magnesium species such as Mg(OH)_2_ forms is present around zeolite particles after cationic exchange (see Section 2.3) as already reported in the literature [36,37,38].

Since the XRF equipment does not detect light atoms such as lithium, the determination of the lithium exchange ratio was performed through Inductive Coupled Plasma Optical Emission Spectroscopy (ICP-OES) analysis, the chemical composition of the raw and lithium exchanged zeolite samples are reported in Table 2.

For LTA-type zeolite, the concomitant sodium loss (Na/Al molar ratio from 1.07 to 0.12) and lithium increase (Li/Al molar ratio from 0 to 0.93) for LiA-1 to LiA-4 samples is an indication of the successful exchange process (Table 2). The same trend is observed with FAU-type zeolite (Table 2).

Unlike magnesium exchange, the overall charge ratio (Na/Al + Li/Al) required in order to compensate the negative charges generated by the presence of aluminum atom and thus maintain the neutrality of the framework is lower than expected. For LTA-type zeolite the values of these ratios are equal to 0.78, 0.75 and 0.89 for LiA-1, LiA-2 and LiA-3, respectively, and to 0.75, 0.87, 0.89 and 0.95 for FAU-type zeolite LiX-1 to LiX-4. This effect seems to be lowered as the number of exchange increases.

Since no impurities are detected by XRD analysis (see Section 3.3.3), the lack of positive charges observed could be linked to the presence of protons not detected by the ICP measurement. These compensating cations could be introduced into the zeolitic framework during the exchange process or the washing steps with slightly acid demineralized water (pH ~ 5.5).

For both zeolites and both cations (lithium and magnesium), increasing the number of exchanges increases the amount of new cations into the zeolitic framework up to approximately 90% after the fourth cationic exchange. In the literature, some studies carried out on zeolite materials mention that a complete cationic exchange is possible [25,39].

**Figure 1:**
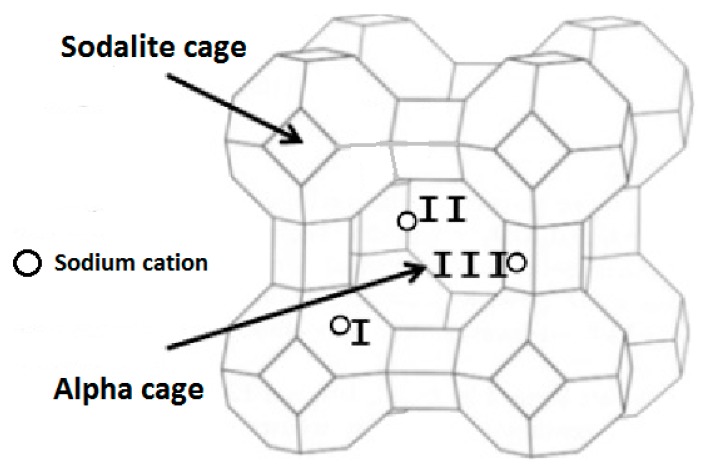
LTA-type zeolite with sodium cations positions.

In this work, the incomplete cationic exchange could be attributed to the lack of exchange steps and/or the difficulty to exchange the sodium cations present in the sodalite cages. Indeed, the sodium cations (1.02 Å) located in the sodalite cages (site I) (Figure 1) are difficult to extract because of the small cage aperture (6MR) with a pore opening of 2.2 [40,41].

Nevertheless, in the case of water adsorption, the main goal of our study, a 100% exchange of Na is useless since water molecules are not adsorbed in sodalite cages. Indeed the kinetic diameter of water molecule (2.65 Å) [42] is larger than the pore opening of sodalite cages.

### 2.2. X-ray Diffraction

**Figure 2:**
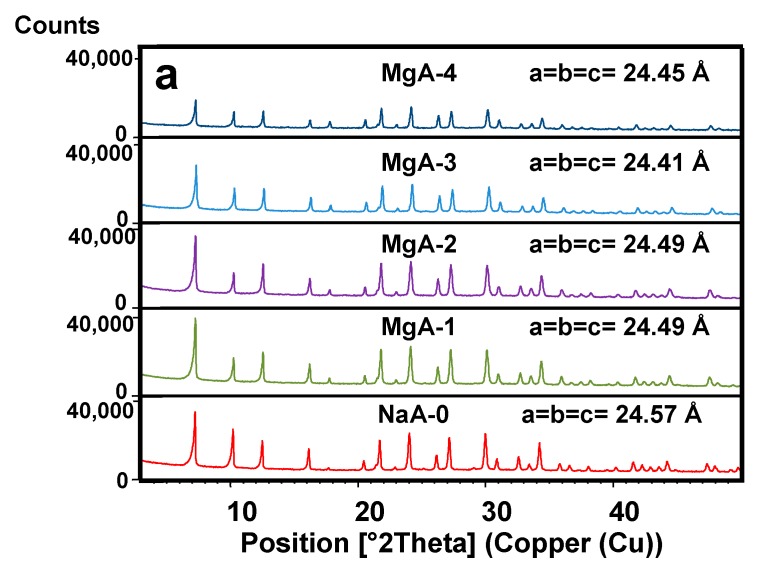

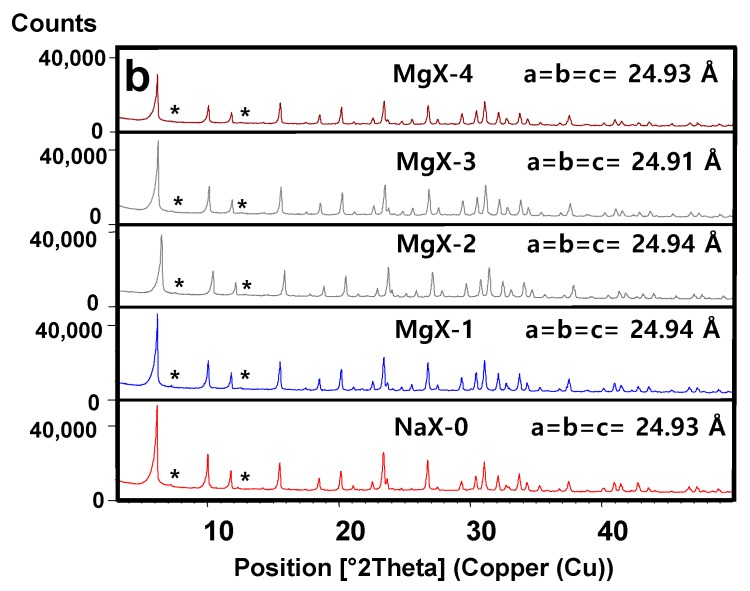
This is a XRD patterns of (**a**) raw and Mg^2+^ exchanged LTA-type zeolite; (**b**) raw and Mg^2+^ exchanged FAU-type zeolite. * Traces of LTA-type zeolites.

**Figure A1:**
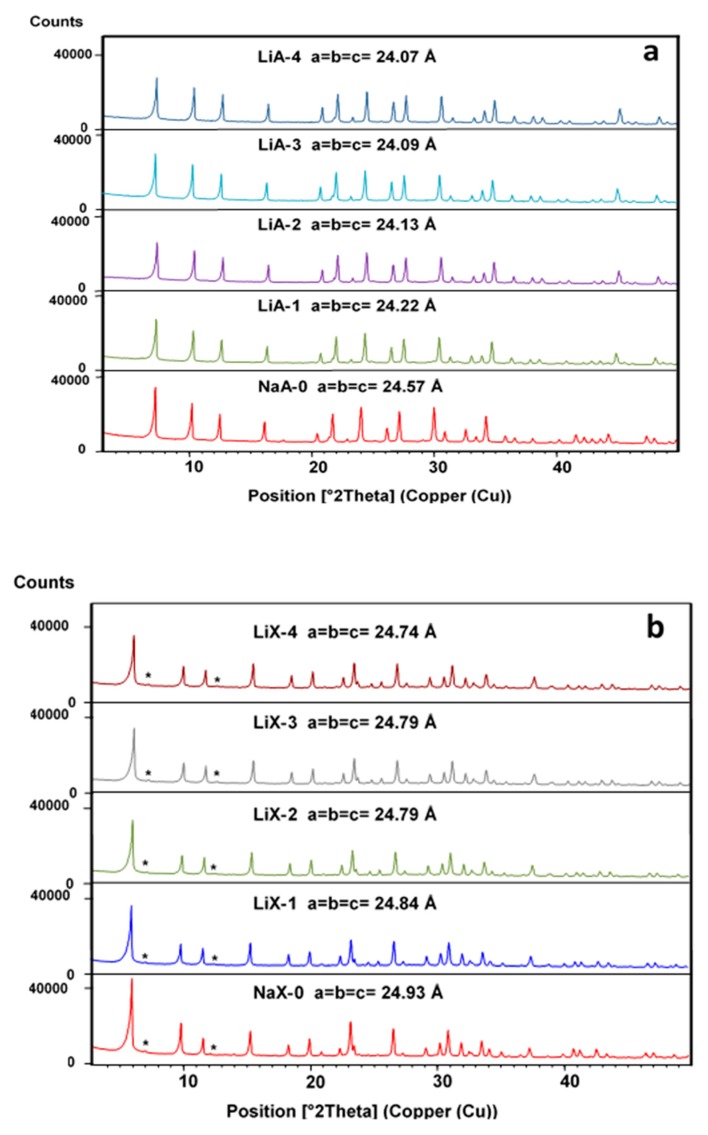
XRD patterns of (**a**) raw and Li+ exchanged LTA-type zeolite and (**b**) raw and Li+ exchanged FAU-type zeolite. * Traces of LTA-type zeolites.

The XRD patterns of both raw and exchanged LTA-type and FAU-type zeolites with Mg^2+^ are displayed in Figure 2a,b. XRD patterns of Li^+^ exchanged samples are available in Appendix A, Figure A1a,b. All samples of LTA zeolite showed a single LTA-type zeolite phase in agreement with the corresponding patterns available in the literature [34] (Pattern 04-016-9920 from ICDD). The unit cell parameters (a, b and c) of the LTA-type zeolite with cubic crystal system and Fm-3c as space group were determined with X’Pert HighScore and STOE Win XPOW softwares [43] according to the Werner algorithm [44]. For the raw LTA material, a = b = c are equal to 24.57 Å, in agreement with the literature [45]. After cationic exchange, the unit cell parameters are a = b = c are equal to 24.49 Å, 24.49 Å, 24.41 Å and 24.45 Å for MgA-1 to MgA-4, respectively and 24.22 Å, 24.13 Å, 24.09 Å and 24.07 Å for LiA-1 to LiA-4, respectively. With more cationic exchange steps, the smaller cation radius of lithium (0.69 Å) and magnesium (0.72 Å) in comparison with sodium (1.02 Å) [46] lead to a structure contraction which is more pronounced for lithium exchanged samples.

In the case of FAU-type zeolite, two phases are observed: FAU-type zeolite as the main product (Pattern 01-070-4281 from ICDD) and traces of LTA. Indeed, in large scale synthesis, LTA is often found as impurity (See Figure 2b). For the raw FAU material, a = b = c are equal to 24.93 Å, in agreement with the literature [45]. After cationic exchange, the unit cell parameters are a = b = c = 24.94 Å, 24.94 Å, 24.91 Å and 24.93 Å for MgX-1 to MgX-4, respectively, and 24.84 Å, 24.79 Å, 24.79 Å and 24.74 Å for LiX-1 to LiX-4, respectively. A more pronounced contraction of the FAU structure is observed after exchange with lithium as in the case of the LTA-type zeolite.

Changes in peaks intensities and slight shifts are observed when sodium cations are replaced by other alkali or alkali earth metal cations. These results were already observed in the literature [47,48,49] and were explained as a consequence of the difference of the scattering power which is specific to each cation and also by a slightly different sites occupation in the pores [49,50].

This is also an indication of a successful cation exchange. Despite those observations, all XRD patterns of exchanged samples are similar to the parent materials indicating that cationic exchange did not affect significantly the structure at the long range.

### 2.3. Scanning Electron Microscopy (SEM) and Energy Dispersive X-rays Spectroscopy (EDX) Characterization

**Figure 3:**
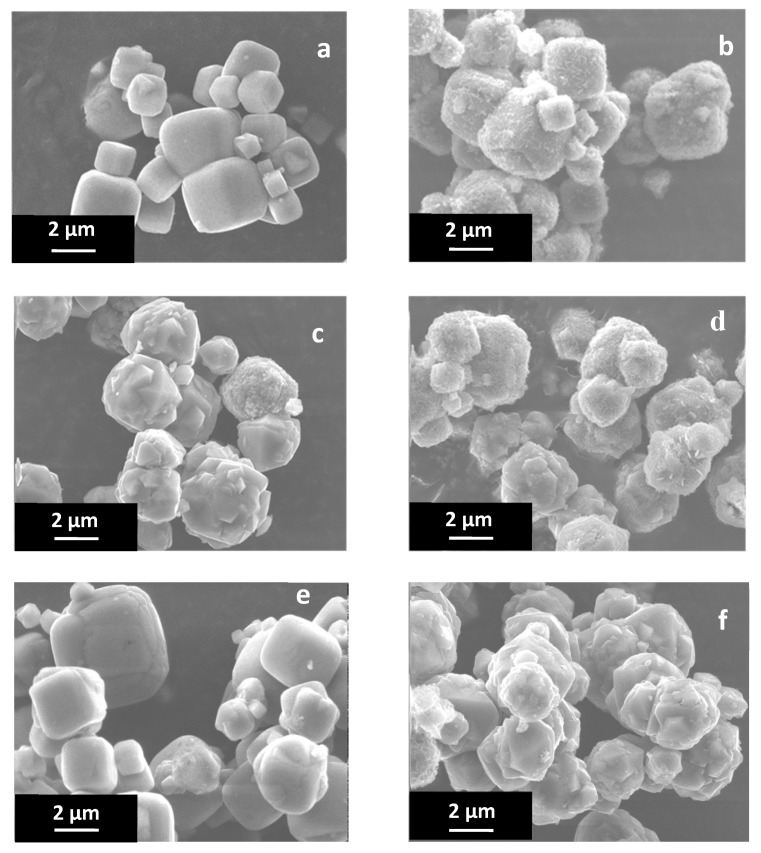
SEM images of (**a**) NaA-0; (**b**) MgA-4; (**c**) NaX-0; (**d**) MgX-4; (**e**) LiA-4 and (**f**) LiX-4 samples.

SEM images of raw materials and exchanged samples (with Li^+^ and Mg^2+^) of LTA-type and FAU-type zeolites are displayed in Figure 3.

Figure 3a shows crystallized phase with cubic morphology characteristic of LTA-type zeolites and a particle size ranging from 1 to 5 µm. Figure 3c shows that the FAU-type zeolite exhibits interconnected bipyramidal crystals with a pseudo-spherical morphology while a bipyramidal morphology is expected. The particle size range is between 1 to 4 µm.

**Figure A2:**
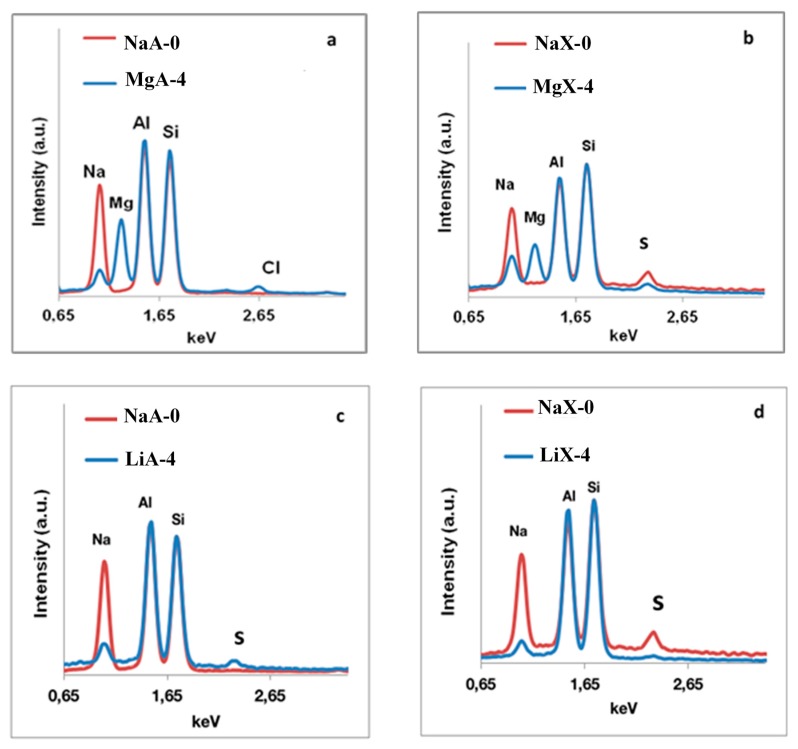
EDX spectra of (**a**) NaA-0 and MgA-4; (**b**) NaX-0 and MgX-4; (**c**) NaA-0 and LiA-4 and (**d**) NaX-0 and LiX-4 samples.

In the case of exchange with magnesium cations for both LTA-type and FAU-type zeolites (see Figure 3), the morphology of the crystals is preserved but small particles seem to cover the crystals surface. XRF and EDX analysis displayed in Table 1 and Figure A2 (Appendix A), respectively, report a loss of the sodium cations after exchange process in favor of the selected new cation Mg^2+^. Figure A2a confirms the presence of chloride anions only for LTA-type zeolite sample after exchange with magnesium as observed by XRF analysis. These chlroride anions can be removed by additional washing with demineralized water.

**Figure 4:**
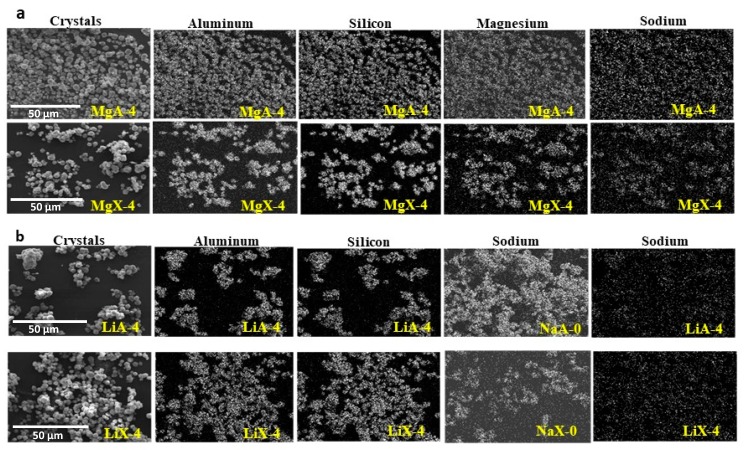
EDX mapping of aluminum, silicon, sodium and magnesium elements present in (**a**) MgA-4, MgX-4 and (**b**) LiA-4, LiX-4 samples and sodium mapping in the raw materials. The scale is similar for all sample series.

The elemental distribution of Si, Al, Na and Mg in the samples was studied using EDX mapping, which is displayed in Figure 3a,b. Each white pixel shows the presence of the corresponding atom. The loss of sodium cations in favor of the exchanged cation (Li or Mg) is confirmed by the loss of intensity (whiteness) between EDX Na mapping shown on exchanged materials compared to the one of raw materials (see Figure 4). Figure 4 shows also a uniform distribution of Mg atoms in the particles which indicate one more time the success of the exchange process. 

As shown by SEM pictures, both LTA-type and FAU-type zeolites exchanged with magnesium cations are covered with small nanoparticles. Unfortunately, EDX analysis is not able to give the composition of this coating because of interference with the zeolite crystals. 

In a recent work of Henao-Sierra et al. on LTA-type zeolite crystals exchanged with nickel and silver cations, additional phase onto the surface were identified [47]. The associated XRD analysis allowed the identification of new crystalline phases composed of NiO or Ag_2_O, respectively.

Bae et al. reported the presence of MgO phase on the surface of MFI-type zeolite [36]. According to their work, MgO transforms in Mg(OH)_2_ upon hydration. SEM photographs describing their MFI-type zeolite coated with Mg(OH)_2_ have similarities with our observations on magnesium exchanged sample of LTA type zeolite. In addition, Koh et al. reported the formation of Mg(OH)_2_ precipitate when MgCl_2_ salt was mixed with basic solution [37,38] during the 4 Å zeolite synthesis. SEM images of our exchanged samples are similar with SEM images observed in the above cited papers. Since our commercial zeolite samples show basic behavior in aqueous solution free of MgCl_2_ (the pH of the solution used for the exchange is also slightly basic), the presence of MgO and/or Mg(OH)_2_ is possible. Nevertheless, because no additional phase is detected by XRD analysis, the amount of this coating must be small.

### 2.4. Solid-State Nuclear Magnetic Resonance (NMR)

**Figure 5:**
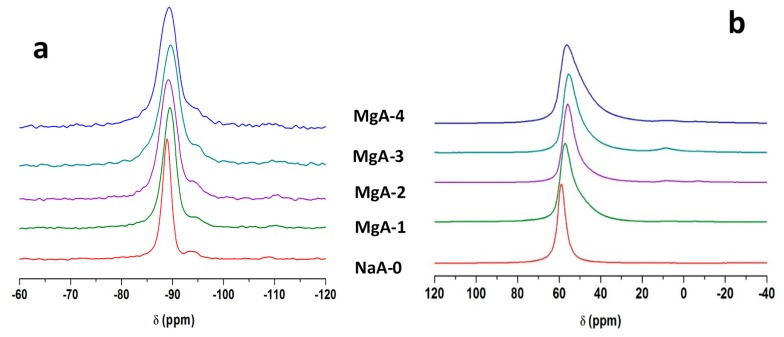
^29^Si-MAS NMR spectra (**a**) and ^27^Al-MAS NMR spectra (**b**) of NaA-0 and its associated MgA-1 to MgA-4 samples. Only the isotropic region is shown.

^29^Si- and ^27^Al-MAS NMR were performed to study the local environments of the corresponding atoms after cationic exchange. ^29^Si-MAS NMR spectra of NaA-0 and the associated MgA-1 to MgA-4 samples are displayed in Figure 5a. One main resonance is detected at −89 ppm corresponding to tetrahedral Si(OAl)_4_ species typical of LTA-type zeolite [51]. An additional resonance is seen around −94 ppm accounting for around 4% of the total signal whatever the sample. Note that this small resonance is already present in the parent material which points toward an impurity in small amount because it is not detected by XRD. This resonance can correspond to Si(OSi)(OAl)_3_ species [52] and can also be related to the presence of extra framework aluminum in the exchanged samples. A broadening of the main signal is observed homogeneously as the exchange rate increases, the width at half height increases from 121 Hz for the raw sample to 247 Hz after four exchanges.

This could result from a distribution of environments around Si atoms. Indeed, since the exchange is not total, both magnesium and sodium cations coexist within the framework. The observed broadening could also result from heteronuclear dipolar interaction between silicon atoms and Mg^2+^ charge compensating cations if both are close [53].

Figure 5b displays ^27^Al-MAS NMR spectra of NaA-0 and the associated MgA-1 to MgA-4 samples. One main resonance is detected at 59 ppm corresponding to tetrahedrally coordinated aluminum Al(OSi)_4_ as expected for LTA-type zeolite [51]. As the exchange rate increases a broadening of the main resonance is observed and the shape of the peak also becomes dissymmetric indicating a distribution of environment in agreement with ^29^Si-MAS NMR results. The ^27^Al-MAS NMR spectra of exchanged samples also present weak signals at 10 ppm and −5 ppm that correspond to octahedral aluminum atoms. As already suggested by ^29^Si-MAS NMR a small (<7% of the total signal) extraction of aluminum atoms from the zeolite framework seems to occur and increases as the exchange rate increases.

**Figure 6:**
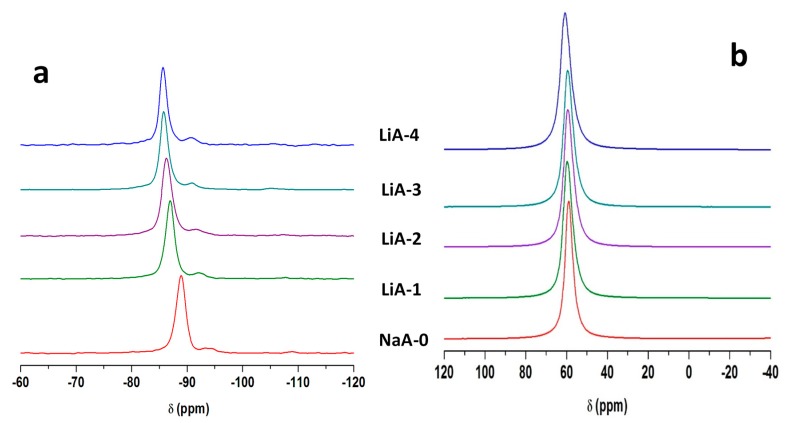
^29^Si-MAS NMR spectra (**a**) and ^27^Al-MAS NMR spectra (**b**) of NaA-0 and its associated LiA-1 to LiA-4 samples. Only the isotropic region is shown.

Figure 6a shows the ^29^Si-MAS NMR spectra of NaA-0 and the associated LiA-1 to LiA-4 samples.

The resonances detected for the MgA samples are also observed for the LiA samples. It is worth noting that no broadening of the main resonance is observed upon Li exchange, but the position of the main resonance is shifted from −89 to −87, −86.5, −86 and −85.6 ppm as the exchange steps progress for NaA-0 and the associated LiA-1 to LiA-4, respectively. This shift, observed for all peaks, is probably related to the contraction of the unit cell as mentioned in the Section 3.3.3. This phenomenon was already observed by Price et al. [49] since ^29^Si chemical shift is known to depend on the bond lengths and bond angles. This effect, linked to the cation size, is more pronounced for Li^+^ whose radius is 0.69 Å than for Mg^2+^ (radius 0.72 Å) instead of 1.02 Å for Na^+^ [46].

Figure 6b displays ^27^Al-MAS NMR spectra of NaA-0 and the associated LiA-1 to LiA-4 samples. A small low field shift (from 59.8 to 60.8 ppm) of the resonance is detected as the exchange rate increases together with a slight broadening (+75Hz) indicating a small modification of the local environment of aluminum atoms but less pronounced than in the case of Mg^2+^ exchange. This observation may be attributed to the nature of the divalent magnesium cation which leads to less required cations into the framework in order to compensate the negative charges generated by the aluminum atoms. Therefore, the local environment of aluminum atoms is less affected after Li exchange than after Mg exchange. It is worth to note that no extra framework aluminum is detected whatever the number of Li exchange. Since the conditions of cationic exchange are identical for magnesium and lithium exchanges, this seems to show that lithium solution has less effect on the zeolite structure.

**Figure 7:**
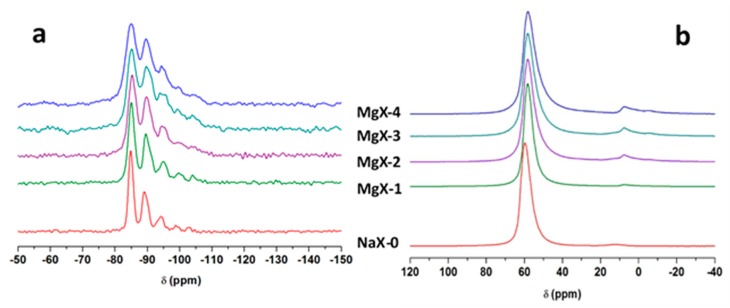
^29^Si-MAS NMR spectra (**a**) and ^27^Al-MAS NMR spectra (**b**) of NaX-0 and its associated MgX-1 to MgX-4 samples. Only the isotropic region is shown.

^29^Si-MAS NMR spectra of NaX-0 and the associated MgX-1 to MgX-4 samples are displayed in Figure 7a.

The spectrum exhibits 5 resonances characteristic of Q^4^ species, located between −85 and −103 ppm ascribed to different Si(Al)_n_ (with n = 0 to 4) species as expected for FAU-type zeolites. The decomposition of each spectrum is reported in Table 3.

The five characteristic resonances observed for the parent material are detected after exchange with magnesium cations. As already mentioned for LTA-type zeolite (see Figure 5), a broadening of the resonances is observed and it is more pronounced when the number of exchanges increases. This suggests a distribution of environments. Because the ^29^Si chemical shift is very sensitive to the local Si, Al ordering in the tetrahedral framework, Si/Al ratio can be calculated from the decomposition of each ^29^Si-MAS NMR spectrum (see Table 3). They are consistent with XRF results (See Table 1) confirming that the characteristic structure of FAU-type zeolite is maintained after exchange. Figure 7b displays ^27^Al-MAS NMR spectra for NaX-0 and the associated MgX-1 to MgX-4 samples. For NaX-0, only one main resonance is detected at 59 ppm corresponding to tetrahedrally coordinated aluminum Al(OSi)_4_ as expected for FAU-type zeolite [51]. As the exchange rate increases a broadening of the main resonance is observed and the shape of the peak also becomes slightly dissymmetric indicating a distribution of environment in agreement with ^29^Si-MAS NMR results. The ^27^Al MAS NMR spectrum of NaX-0 samples displayed in Figure 7b shows a weak resonance around 12 ppm, accounting for 1% of the total signal, which corresponds to octahedral aluminum atoms. This observation seems not surprising for zeolites from industrial batches. Cationic exchange seems to favor the formation of octahedral aluminum atoms with weak signals detected between 15 ppm and −5 ppm accounting for 3%, 10%, 13% and 6% of the total signal for MgX-1 to MgX-4 samples, respectively. As already suggested by the small increase of Si/Al ratio, a small extraction of aluminum atoms from the zeolite framework seems to occur as the exchange rate increases from 1 to 3. Surprisingly the MgX-4 sample exhibits a similar Si/Al ratio and similar amount of extra framework species than the parent sample. This could be due to variations during the washing process.

**Figure 8:**
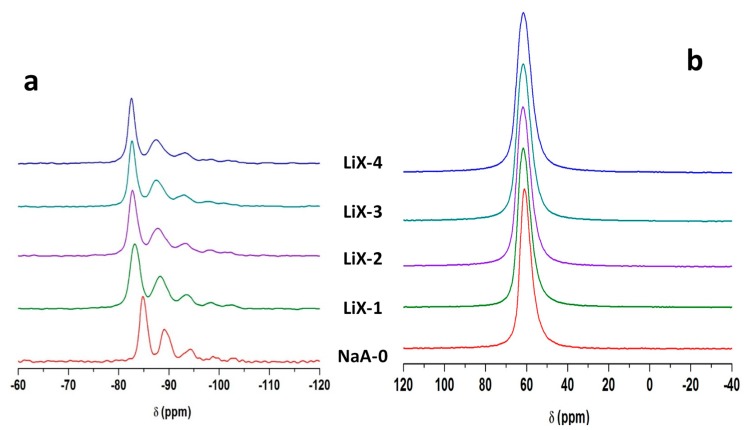
^29^Si-MAS NMR spectra (**a**) and ^27^Al-MAS NMR spectra (**b**) of NaX-0 and its associated LiX-1 to LiX-4 samples. Only the isotropic region is shown.

Figure 8a shows the ^29^Si-MAS NMR spectra for NaX-0 and the associated LiX-1 to LiX-4 samples.

The resonances detected for the MgX samples are also observed for the LiX samples. The spectra exhibit 5 resonances characteristic of Q^4^ species, detected between −82.6 ppm and −100 ppm and ascribed to different Si(Al)_n_ species (n = 0 to 4). It is worth noting that no broadening of the main resonance is observed upon Li exchange, but the position of the main resonance is shifted from −85 to −83.2, −82.8, −82.7 and −82.6 ppm as the exchange steps progress for NaX-0 and the associated LiX-1 to LiX-4 samples, respectively. This shift, observed for all peaks, is probably related to the contraction of the unit cell as already mentioned in the Section 3.3.3. The respective Si/Al ratio is shown in Table 4. They are consistent with those observed thanks to XRF measurements (See Table 1). Figure 8b displays ^27^Al-MAS NMR spectra of NaX-0 and the associated LiX-1 to LiX-4 samples. A unique resonance at 59 ppm is detected similar to the main resonance of MgX samples. A slight broadening is observed as the cationic exchange steps increase as already mentioned for LiA samples. No extra framework aluminum is detected.

^29^Si- and ^27^Al-MAS NMR spectra are sensitive to cationic exchange of Na^+^ by Li^+^ or Mg^2+^ indicating different silicon and aluminum environments. However, despite slight modifications of the local order, the resonances characteristic of LTA-type and FAU-type zeolites are observed in agreement with ICP, XRF and XRD measurements.

### 2.5. N_2_ Adsorption-Desorption Isotherms Characterization 

**Figure 9:**
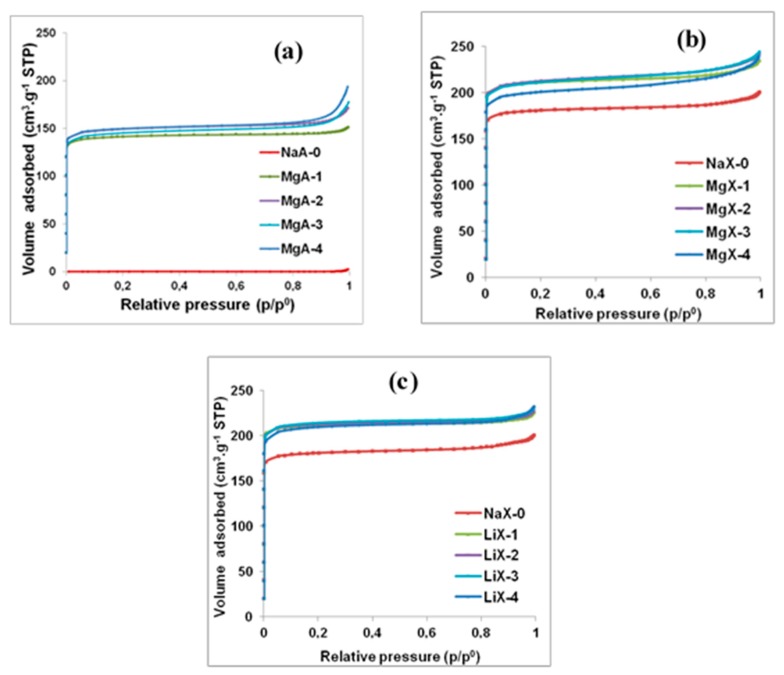
Nitrogen adsorption isotherms of raw and Mg^2+^ exchanged LTA (**a**) and FAU (**b**) type zeolites and (**c**) Li^+^ exchanged FAU-type zeolites.

**Figure A3:**
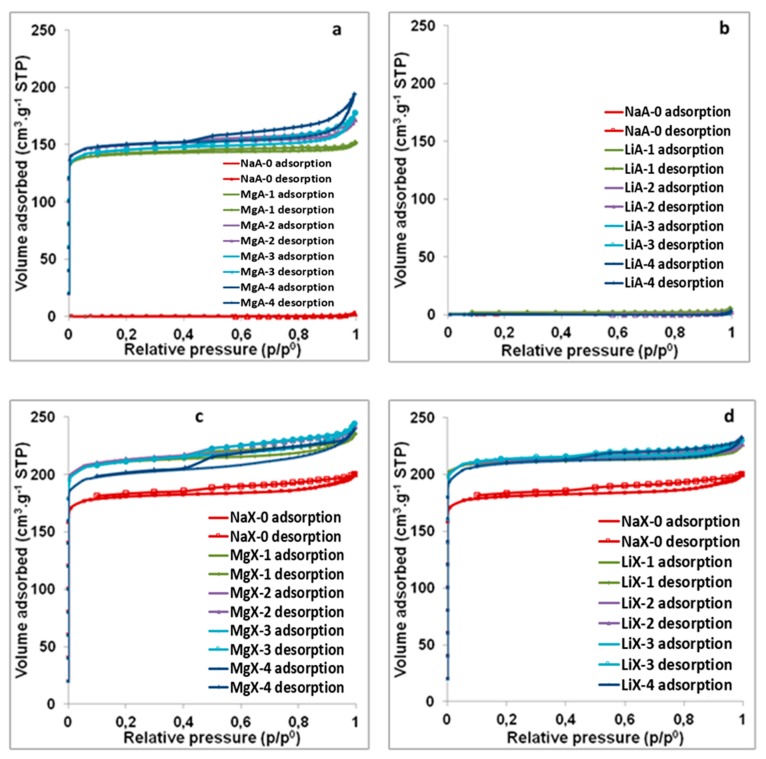
Nitrogen adsorption-desorption isotherms for raw and Mg^2+^ exchanged LTA (**a**) and FAU (**c**) type zeolites and Li^+^ exchanged LTA (**b**) and FAU (**d**) type zeolites.

The nitrogen adsorption isotherms of the raw zeolites and exchanged zeolites are displayed in Figure 9 and the complete nitrogen sorption isotherms are shown in Figure A3.

The textural properties (BET surface, microporous volume) of all these samples are shown in Table 5. The adsorption isotherm of the sodium form of LTA-type zeolite is displayed in Figure 9a. As expected, no N_2_ adsorption is observed. This phenomenon was already mentioned in the literature [39,54,55,56] and is due to the position of Na^+^ cations near to the pore opening which obstruct the accessibility of N_2_ to the microporosity.

In contrast, MgA-1, 2, 3 and 4 samples (Figure 9a) allow nitrogen diffusion through the porosity of LTA-type zeolite and exhibit a type I isotherm according to the IUPAC classification of isotherms [57]. The large adsorption capacity observed at p/p^0^ = 0.1 for all samples shows an ability to adsorb nitrogen even as traces. Non-significant differences are observed on the adsorption capacities (around 140 cm^3^ g^−1^, microporous volume of 0.21 to 0.22 cm^3^ g^−1^) of N_2_ while increasing the number of steps of Mg^2+^ exchange on LTA-type zeolite indicating that only one exchange is enough to access to the highest microporous volume. This first exchange corresponds to the replacement of 59% of the sodium initially present. Since the samples were in powder forms, the adsorption observed between p/p^0^ = 0.9 and 1 is attributed to the inter-particular porosity.

The N_2_ adsorption isotherm of NaX-0 zeolite (Figure 9b) show a nitrogen adsorption capacity around 170 cm^3^ g^−1^ (microporous volume of 0.27 cm^3^ g^−1^) which is lower than the one expected for FAU-type zeolite (around 0.32 to 0.34 cm^3^ g^−1^) [56,58]. The LTA impurities contained in the raw material (see Figure 2b) could explain this lower value. When samples are exchanged with magnesium a capacity up to 210 cm^3^ g^−1^ representing an increase of 24% of the adsorbed volume, is observed compared to the raw sample while an increase of 15% of the microporous volume is noticed (0.27 to 0.31 cm^3^ g^−1^). For all samples, the BET surface increases with the microporous volume, to around 550–600 m^2^ g^−1^ for LTA-type zeolite samples and from 738 m^2^ g^−1^ for raw FAU-type zeolite sample to around 860 m^2^ g^−1^ for magnesium exchanged samples.

For LTA samples, the increase of the adsorbed nitrogen amount between the sodium form and the magnesium form can be explained by the cations positions displayed in Figure 1 [55,59]. Indeed, monovalent cations such as sodium are located as follow: 67% occupy the site I at the center of the six member ring corresponding to the window between the sodalite cage and the alpha cage, 25% occupy the site II near the plane of the eight membered ring representing the aperture of the porosity and 8% are in site III near the four-ring inside the cavity [55,59]. It is essential to note that the porosity exists; it is only the N_2_ probe molecule which cannot access it.

Indeed, the eight membered pore opening of LTA-type zeolite is about 4.21 Å without any compensating charge cation according to the International Zeolite Association (IZA). Located at site II, sodium cations with a diameter of about 2 Å obstruct partially the pore opening. Thus nitrogen with a kinetic diameter of about 3.6 Å is not be able to enter the microporosity of NaA as mentioned earlier. The same behavior is observed on the LTA-type zeolite exchanged with lithium cation (see Figure A3).

When monovalent cations are exchanged by bivalent cations, the cations’ locations change. A modeling study showed that in the calcium form of the LTA-type zeolite, no cations were placed at site II representing the aperture of the porosity, the site I inside the six-membered ring seems to be preferred [60]. The absence of cations at the aperture leads to a pore opening of around 4.21 Å according to the International Zeolite Association (IZA) allowing free diffusion of nitrogen molecules. Consequently, after sodium exchange with magnesium, nitrogen molecules can reach the porosity. 

FAU-type zeolite consists of sodalite building blocks joined tetrahedrally via double six-rings (D6R) creating a supercage (large cavity) in the center of the unit cell with an aperture delimited by a 12 membered ring [39]

The cations are usually located as followed [39,41]: site I at the center of the D6R, site I’ in the sodalite cavity near the D6R ring, site II in the super cage near the 6 ring unit (S6R), site II’ in the sodalite cage near the 6 ring unit (S6R), site III and III’ in the super cage facing the 4 ring unit. Monovalent cations such as sodium are usually located in site II and III in hydrated zeolite X [41,61,62]. It is well known that after dehydration compensating cations migrate between different cation locations into the structure in order to maximize their coordination [61]. Indeed, the literature mentioned that after dehydration Mg^2+^ cations (Zeolite MgX) are located at site I and II instead of site I’, II and III for the hydrated form [61]. For both types of zeolite samples, two reasons could explain the uptake increase. The global porous volume of the zeolite stays unchanged, depending on the amount and kinetic diameter of the compensating cation, the available microporous volume will be modified. In case of bivalent cations, less cations are necessary to compensate the negative charges generated by the presence of aluminum in the zeolite framework. In addition, the lower radius of Mg^2+^ cations (0.72 Å) compared to Na^+^ (1.02 Å) implies a lower occupied space. Others parameters should also be taken into account namely interactions between the charge compensating cation and the adsorbate (as it will be shown in water adsorption part). All these parameters allow an increase of the available microporous volume and adsorption capacities (see Table 5).

When FAU-type zeolite samples are exchanged with lithium, see Figure 9c, a capacity of 205 cm^3^ g^−1^ to 210 cm^3^ g^−1^ (microporous volume of 0.32 to 0.33 cm^3^ g^−1^) representing an increase of 21% to 24% of the adsorbed volume and 19% to 22% of the microporous volume is observed in comparison with the raw sample NaX-0. This increase is assigned to the decrease of the lithium radius (0.69 Å) with respect to the sodium one, increasing the available microporous volume.

Since less bivalent magnesium cations are required to compensate the negative charges generated by the presence of aluminum in the zeolite framework, the microporous volume of the magnesium exchanged samples was expected to be higher than those exchanged with lithium. For lithium exchanged samples only the size factor (Na^+^ 1.02 Å versus Li^+^ 0.69 Å) contributes to the increase of the available microporous volume while magnesium exchanged samples take the advantage of both reduction of required number of cations and a lower size. However, lithium cations have a lower size than magnesium cations (decrease of 4%): 0.69 Å and 0.72 Å respectively.

Similar microporous volume observed for both magnesium and lithium FAU exchanged samples (~0.31–0.33 cm^3^ g^−1^) (Table 5) seems to point that the gain resulting from the smaller size of lithium cation partially compensate the gain resulting from having less magnesium cations into the framework. The increase of the accessible porous volume after cationic exchange especially with Mg^2+^ should allow increasing the performances of those materials regarding water adsorption.

### 2.6. H_2_O Adsorption Isotherms Characterization 

**Figure 10:**
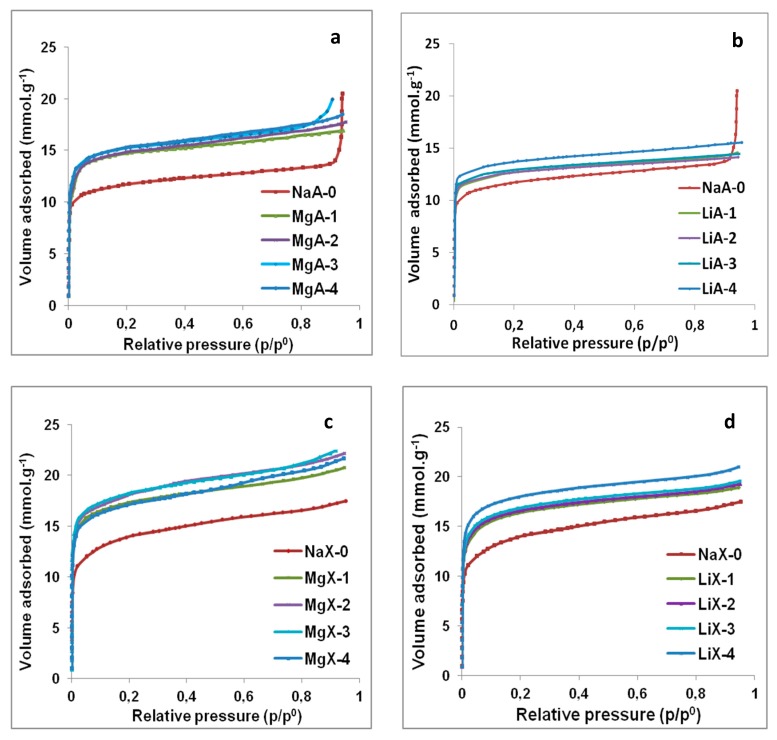
Water adsorption isotherms for raw and Mg^2+^ exchanged LTA (**a**) and FAU (**c**) type zeolites and Li^+^ exchanged LTA (**b**) and FAU (**d**) type zeolites.

The water adsorption isotherms of the raw materials and the Li^+^ and Mg^2+^ exchanged LTA-type and FAU-type zeolites are displayed in Figure 10. The water adsorption capacities were determined at p/p^0^ = 0.2 (representing the adsorption in the microporosity of the samples) and reported for each sample in Table 5.

According to Figure 10, water adsorption is observed for each zeolite sample whatever the nature and the charge compensating cation. The collected data for all samples show type I isotherms according to IUPAC classification [57]. Water adsorption capacities obtained for the raw LTA-type and FAU-type samples (~21.1 Wt.% for LTA and ~25.3 Wt.% for FAU) displayed in Table 5 are deduced from the molar capacity and are in agreement with the literature [29,58]. A gain of the water adsorption capacity is observed for each exchanged sample in comparison with their associated raw samples, in agreement with nitrogen adsorption analysis.

According to Table 5, the NaA-0 sample has a water adsorption capacity of 21.1 Wt.%. When the samples are exchanged with magnesium, the capacity increases from 26.5 Wt.% (MgA-1) to 27.5 Wt. % (MgA-4) representing an increase of 26% to 30% of the adsorbed volume in comparison with NaA-0. It is worth to note that increasing the number of exchange step from one to four does not increase significantly the water adsorption capacity. In Figure 10a,b, the adsorption observed between p/p^0^ = 0.9 and 1 is attributed to the inter-particular porosity. From the water adsorption isotherm of NaX-0 zeolite displayed in Figure 10c, a water adsorption capacity of 25.3 Wt.% is deduced (see Table 5). When the samples are exchanged with magnesium, the water capacity increases from 31.2 Wt.% (MgX-1) to 32.8 Wt. % (MgX-3) i.e. an increase of 23% to 30% of the adsorbed water in comparison to NaX-0. For lithium exchanged LTA samples, a water adsorption capacity of 24.7 Wt.% for LiA-4 sample is observed while exchanges 1, 2 and 3 show adsorption capacities of 22.8 Wt.%, 22.9 Wt.% and 23.3 Wt.% respectively, see Table 5. The global water adsorption increases between 8% and 17% in comparison with NaA-0. The lithium form of FAU-type zeolite displayed in Figure 10d shows a water adsorption capacity of 29.5 Wt.% for LiX-1 while exchanges LiX-2, LiX-3 and LiX-4 show adsorption capacities of 29.9 Wt.%, 30.4 Wt.% and 32.4 Wt.%, respectively. The global water adsorption increases between 17% and 28% which indicates that using lithium for cationic exchange with LTA-type or FAU-type zeolites is less performant than magnesium for water adsorption optimization.

**Figure 11:**
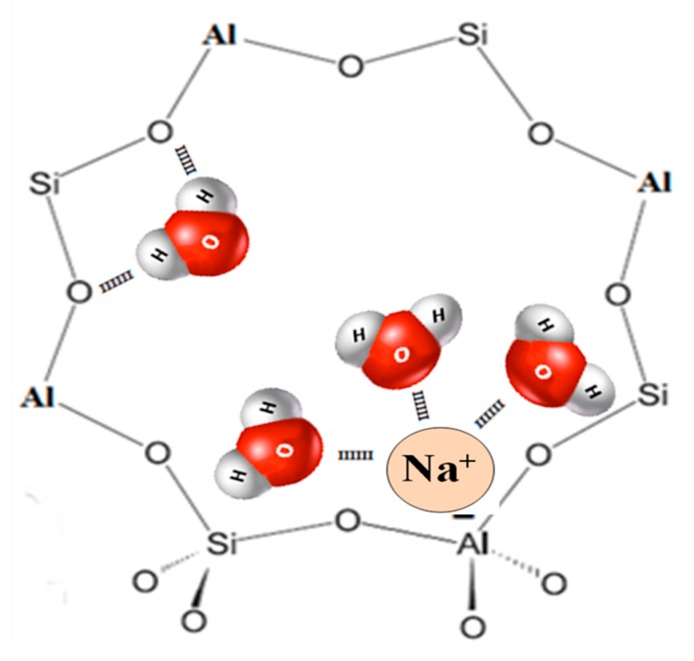
Water interactions into hydrophilic zeolites framework, example of LTA-type zeolite. Scheme representing the 8-membering aperture of the LTA-type zeolite, the elemental composition (Si, Al, O, cations) is homogeneous in all the crystal leading to the same adsorption properties.

Water adsorption into hydrophilic zeolite such as LTA-type and FAU-type depends mainly on the available porous volume, interactions with oxygen atoms of the framework and interactions with the compensating cations (solvating layer). Since hydrophilic zeolite frameworks and water molecules are polarized, electrostatic attractions can occur. An illustration of the possible interactions between zeolite framework and water molecules is displayed in Figure 11.

These results show that modifying the charge compensating cations of LTA-type and FAU-type zeolites change the water adsorption behavior. Water adsorption capacities improve up to 30% of the adsorbed volume for LTA-type and FAU-type zeolites exchanged with magnesium, up to 24% for LTA-type zeolite exchanged with lithium and up to 28% for FAU-type zeolite exchanged with lithium. Lithium exchanged samples show interesting increase of the water adsorbed volume compared to the raw sample. Magnesium as compensating cation shows the higher water adsorption capacities despite the surface coating of the particles.

Since the available microporous volume determined from N_2_ adsorption is similar for lithium and magnesium exchanged FAU samples (see Table 5) while water adsorption is higher for magnesium exchanged samples, the nature of the interactions between the charge compensating cations and water molecules should be considered. Indeed, bivalent magnesium cations possess a higher polarization charge which implies stronger interactions with water and thus higher hydration layer than monovalent lithium cation [46,63].

## 3. Materials and Methods 

### 3.1. Raw Materials

LTA-type zeolite (NaA) and FAU-type zeolite (NaX) were provided in powder form by Aptar CSP Technologies (Niederbronn-Les Bains, France). Lithium chloride (LiCl, ACS Reag. Ph. Eur >99%) and magnesium chloride (MgCl_2_·6H2O, ACS-ISO for analysis >99%) salts were purchased from Merck (Saint Quentin Fallavier, France) and Carlo Erba (Val-de-Reuil, France), respectively, and used for the ion exchange processes.

### 3.2. Cation Exchange

NaA and NaX zeolites were modified by exchanging the sodium compensating cations present in the parent zeolites with magnesium (Mg^2+^) or lithium (Li^+^) cations by a cationic exchange process in MgCl_2_ and LiCl aqueous solution. The raw zeolite (20 g) was blended with 1 M aqueous cationic solution that was prepared by mixing LiCl salt (16.96 g) or MgCl_2_ salt (81.32 g) with 400 mL of demineralized water. The reaction mixture was then heated at 80 °C for 2 h under stirring. The mass ratio of the reaction mixture is 1 g of zeolite for 20 mL of electrolyte aqueous solution. The pH value of this mixture is between 7 and 9. Zeolites were then filtered by centrifugation (8000 rpm, 5 min) and washed 3 times under stirring (10 min) with cold demineralized water (~200 mL). All the samples were then dried during 24 h minimum, at 80 °C. The cationic exchange process was repeated up to 4 times. After each cationic exchange the samples were fully characterized. The exchange ratio of samples was determined by X-Ray Fluorescence (XRF). The obtained exchanged zeolites were denoted as follows: cA-y or cX-y with c the major compensating cation and y is the number of exchange experiment. For example MgA-2 means zeolite A exchanged two times with MgCl_2_ aqueous solution.

### 3.3. Characterization Techniques

#### 3.3.1. X-ray Fluorescence (XRF)

Chemical analyses were performed using an X-Ray Fluorescence (XRF) spectrometer (Zetium, 4 kW, PANalytical, Limeil-Brévannes, France) on samples previously pressed into 13 mm diameter pellets for 10 minutes at a pressure of 5 tons.

#### 3.3.2. Inductive Coupled Plasma Optical Emission Spectroscopy (ICP-OES)

The samples underwent acid digestion at room temperature for 24 h (0.05 gr of sample are added to 3 mL of 48.9% hydrofluoric acid (HF)). The solution thus obtained is diluted to 30 mL with distilled water and then filtered through a 0.45 μm filter before analysis using an ICAP 6300 DUO instrument (Thermo, Villebon-sur-Yvette, France).

#### 3.3.3. X-ray Diffraction (XRD)

The X-Ray diffraction patterns were recorded on a PANalytical MPD X’Pert Pro diffractometer (Limeil-Brévannes, France) operating with Cu Kα radiation (Kα = 0.15418 nm) equipped with an X’Celerator real-time multiple strip detector (active length = 2.12 °2θ). The XRD powder patterns were collected at 22 °C in the 3° < 2θ < 50° range, by step of 0.017° in 2θ and with a time of 220 s by step.

#### 3.3.4. Scanning Electron Microscopy (SEM) and Energy Dispersive X-rays Spectroscopy (EDX)

Scanning Electron Microscopy micrographs and Energy Dispersive X-Rays spectroscopy maps were obtained on an XL 30 FEG microscope (Verdun, France). Before analysis, the samples were coated with a fine carbon layer using a SCD004 sputter coating system (BAL-TEC (LEICA MICROSYSTEMES SA), Nanterre, France) in order to improve the electrical conductivity.

#### 3.3.5. Solid-State Nuclear Magnetic Resonance (Solid-State NMR)

^29^Si solid-state Magic Angle Spinning (MAS) NMR spectra with ^1^H decoupling were recorded on an AVANCE II 300WB spectrometer (B0 = 7.1 T, Bruker, Wissembourg, France) operating at 59.59 MHz with a 2.4 µs pulse duration corresponding to a flip angle of π/6 and 80 s of recycling delay. Samples were packed in a 7 mm cylindrical zirconia rotor and spun at a spinning frequency of 4 kHz. ^29^Si chemical shifts were referenced to tetramethylsilane (TMS). ^27^Al-MAS NMR spectra were recorded on a Bruker AVANCE II 400WB spectrometer (B0 = 9.4 T) operating at 104.2 MHz using a 4 mm cylindrical zirconia rotor and spun at a spinning frequency of 12 kHz. ^2^7Al chemical shifts were given relative to an aqueous solution of aluminum nitrate (Al(NO_3_)_3_). Typical acquisition parameters included a pulse duration of 0.5 μs corresponding to a flip angle of π/12 and 1 s recycle delay. Decompositions of the NMR spectra to extract the proportion of the corresponding species were performed with the DMfit software [64].

#### 3.3.6. N_2_ Adsorption-Desorption Measurements 

The textural characteristics of raw and exchanged zeolite samples were determined from the N_2_ adsorption-desorption isotherms performed at −196 °C using an ASAP2420 instrument (Micromeritics, Merignac, France). Prior to the sorption measurement the samples (50–100 mg) were outgassed under vacuum at 90 °C for 1 h and 300 °C for 15 h to remove the physisorbed water. The Brunauer-Emmett-Teller specific surface area (S_BET_) was calculated by using the BET method while the t-plot method was used to determine the sample microporous volume (V_m_).

#### 3.3.7. Water Adsorption Measurement

Water adsorption isotherms of raw and exchanged zeolite samples were performed at 25 °C using a Micromeritics ASAP 2020 instrument. Prior to the water adsorption measurements, water (analyte) was flash frozen under liquid nitrogen and then evacuated under dynamic vacuum at least 5 times to remove any gases in the water reservoir. The samples (50–100 mg) were outgassed under vacuum at 90 °C for 1 h and 300 °C for 24 h to remove the physisorbed water. The water adsorption capacity of the samples was determined from the water adsorption isotherms.

## 4. Conclusions

In this work, the characterization of LTA-type and FAU-type zeolites exchanged with lithium and magnesium cations were performed. X-ray Fluorescence (XRF), Inductive Coupled Plasma Optical Emission Spectroscopy (ICP-OES) and Energy Dispersive X-Ray spectroscopy (EDX) reported that a significant amount of the new introduced cation is homogeneously distributed into the crystals, sign of a successful cationic exchange. Scanning electron microscopy photographs highlight the cubic morphology of LTA and the incomplete bypiramidal morphology of FAU-type zeolites while XRD and NMR analysis confirmed that the exchange does not lead to significant structural changes. A coating observed on the surface of zeolites exchanged with MgCl_2_ solution is supposed to consist in MgO and/or Mg(OH)_2_. Since no detection of these species has been possible by XRD, the amount is considered as negligible. The replacement of sodium cations by a smaller monovalent cation such as lithium or a smaller bivalent cation such as magnesium leads to the increase of the available microporous volume (+15% and +22% of available microporous volume for FAU-type zeolite exchanged with magnesium and lithium, respectively, compared to the raw sample). More available microporous volume and less congested pore opening, increase the accessibility (N_2_ in LTA) to the micropores and improve the storage of more host molecules such as for example water. In addition, bivalent cations lead to higher degree of ordered water molecules around them, a better spatial organization contributes also to improve water adsorption [65,66]. According to the water adsorption isotherms results, exchanged zeolites with lithium and magnesium showed higher water adsorption capacities than their associated raw samples. The most significant increase of water adsorption is observed for the magnesium exchanged samples (+30% of the adsorbed volume), for both LTA-type and FAU-type zeolites (in comparison with the raw samples). 

Modification of the water adsorption properties of zeolites by cationic exchange process represent one of the easiest methods to implement at the industrial scale since no drastic changes on the material are necessary. Water and salt as main driving force for exchange are sufficient to improve the zeolite adsorption performances. A significant water adsorption increase after only one step of exchange with magnesium cation makes the cationic exchange process viable for industrial applications. Improvement of global water uptake or flexibility regarding water adsorption capacities could be very valuable when applications require limited or lower zeolite amounts.

## Figures and Tables

**Table 1:** Chemical composition of LTA-type and FAU-type zeolites and of their associated magnesium forms determined by XRF (Si/Al, Na/Al, Mg/Al and Cl/Al Molar ratio).

Samples Exchanged with Magnesium	Molar Ratio ^1,2^				Global Charge Ratio
	Si/Al	Na/Al	Mg/Al	Cl/Al	(Na + 2 Mg)/Al
NaA-0	0.97	1.07	0	0	1.07
MgA-1	0.97	0.44	0.34	0.08	1.12
MgA-2	1.04	0.36	0.42	0.07	1.20
MgA-3	1.06	0.22	0.49	0.07	1.20
MgA-4	1.03	0.15	0.50	0.06	1.15
NaX-0	1.23	1.06	0	0	1.06
MgX-1	1.23	0.42	0.32	0	1.06
MgX-2	1.33	0.36	0.40	0	1.16
MgX-3	1.34	0.26	0.48	0	1.22
MgX-4	1.29	0.20	0.48	0	1.16

^1^ Experimental error 3%, ^2^ The ratios are corrected from the slight amount of extra framework aluminum (See Section 2.4).

**Table 2:** Chemical composition of LTA-type and FAU-type zeolites and of its associated lithium form determined by ICP-OES (Si/Al, Na/Al and Li/Al Molar ratio).

Samples Exchanged with Lithium	Molar Ratio ^1^		Global Charge Ratio	
	Si/Al	Na/Al	Li/Al	(Na + Li)/Al
NaA-0	1.04	1.07	0	1.07
LiA-1	1.11	0.41	0.37	0.78
LiA-2	1.14	0.15	0.60	0.75
LiA-3	1.07	0.13	0.76	0.89
LiA-4	1.00	0.12	0.93	1.05
NaX-0	1.25	0.95	0	0.95
LiX-1	1.36	0.34	0.41	0.75
LiX-2	1.33	0.16	0.71	0.87
LiX-3	1.32	0.14	0.75	0.89
LiX-4	1.27	0.11	0.84	0.95

^1^ Experimental error 5%.

**Table 3:** Relative peak area (%) and Si/Al ratio determined from the ^29^Si MAS NMR spectra of the raw and magnesium forms of FAU-type zeolites.

Samples	Q^4^ Si(Al)_4_ (%)	Q^4^ Si(Al)_3_ (%)	Q^4^ Si(Al)_2_ (%)	Q^4^ Si(Al)_1_ (%)	Q^4^ Si(Al)_0_ (%)	Si/Al ^1^
NaX-0	50	30	14	3	3	1.25
MgX-1	43	38	8	8	3	1.29
MgX-2	37	38	16	6	3	1.28
MgX-3	43	30	13	7	7	1.36
MgX-4	49	22	18	6	5	1.27

^1^ Si/Al = $\frac{Si\left(Al\right)4\;+\;Si\left(Al\right)3+\;Si\left(Al\right)2\;+Si\left(Al\right)1+\;Si\left(Al\right)0}{1\;\left(Si\left(Al\right)4\right)+0.75\;\left(Si\left(Al\right)3\right)+0.5\;\left(Si\left(Al\right)2\right)+0.25\;\left(Si\left(Al\right)1\right)}$.

**Table 4:** Relative amount of Q^4^ units (%) and Si/Al ratio determined from the ^29^Si-MAS NMR spectra of the raw and lithium forms of FAU-type zeolites.

Samples	Q^4^ Si(Al)_4_ (%)	Q^4^ Si(Al)_3_ (%)	Q^4^ Si(Al)_2_ (%)	Q^4^ Si(Al)_1_ (%)	Q^4^ Si(Al)_0_ (%)	Si/Al ^1^
NaX-0	50	30	14	3	3	1.25
LiX-1	47	34	12	5	2	1.25
LiX-2	45	36	12	5	2	1.26
LiX-3	42	37	14	5	2	1.28
LiX-4	48	32	14	3	3	1.25

^1^ Si/Al = $\frac{Si\left(Al\right)4\;+\;Si\left(Al\right)3+\;Si\left(Al\right)2\;+Si\left(Al\right)1+\;Si\left(Al\right)0}{1\;\left(Si\left(Al\right)4\right)+0.75\;\left(Si\left(Al\right)3\right)+0.5\;\left(Si\left(Al\right)2\right)+0.25\;\left(Si\left(Al\right)1\right)}$.

**Table 5:** Rate of exchange (Negative charges of the framework compensated by the new cation), BET surface area (S_BET_), microporous volume (V_m_) and water adsorption capacity for LTA-type and FAU-type zeolites modified by Li^+^ and Mg^2+^ ions.

Samples	Sodium Cation Exchange Rate (%)	S_BET_^3^ (m^2^·g^−1^)	V_m_^4^ (cm^3^·g^−1^)	Water Adsorption Capacity ^5^ (mmol g^−1^)	Water Adsorption Capacity ^6^ (Wt.%)
NaA-0	0	x	x	11.7	21.1
MgA-1	59 ^1^	577	0.22	14.7	26.5
MgA-2	66 ^1^	605	0.22	14.9	26.8
MgA-3	79 ^1^	583	0.21	15.2	27.4
MgA-4	86 ^1^	605	0.22	15.3	27.5
NaX-0	0	738	0.27	14.0	25.3
MgX-1	60 ^1^	863	0.31	17.3	31.2
MgX-2	66 ^1^	862	0.31	18.1	32.6
MgX-3	75 ^1^	854	0.31	18.2	32.8
MgX-4	81 ^1^	805	0.30	17.2	31.0
LiA-1	61 ^2^	x	x	12.7	22.8
LiA-2	86 ^2^	x	x	12.7	22.9
LiA-3	88 ^2^	x	x	12.9	23.3
LiA-4	89 ^2^	x	x	13.7	24.7
LiX-1	64 ^2^	877	0.33	16.4	29.5
LiX-2	83 ^2^	862	0.33	16.6	29.9
LiX-3	85 ^2^	866	0.33	16.9	30.4
LiX-4	88 ^2^	841	0.32	18.0	32.4

^1^ Value determined by XRF measurement. Determined from Na/Al ratio (See Table 1), ^2^ Value determined by ICP-OES measurement. Determined from Na/Al ratio (See Table 2), ^3^ Value determined by the BET method, ^4^ Value determined by the the t-plot method, ^5^ Value determined from water adsorption isotherm, ^6^ Value obtained by multiplying the amount of adsorbed water in mmol/g by the molecular weight of water. X = Not porous to nitrogen molecule.

## Appendix A

The sulfur shown on EDX spectra is assigned to impurities of the analysis support (tape).
